# Assessment of the Integrity and Function of Human Term Placental Explants in Short-Term Culture

**DOI:** 10.3390/mps7010016

**Published:** 2024-02-15

**Authors:** Carolina López-Guzmán, Ana María García, Paula Marín, Ana María Vásquez

**Affiliations:** 1 Grupo Malaria, Calle 62 # 52-59 Torre 1 Laboratorio 610, Facultad de Medicina, Universidad de Antioquia, Medellín 050001, Colombia; carolina.lopezg@udea.edu.co (C.L.-G.); ana.garcia6@udea.edu.co (A.M.G.); paulaa.marin@udea.edu.co (P.M.); 2 Escuela de Microbiologia, Calle 67 # 53-108, Bloque 5, Oficina 5-135, Universidad de Antioquia, Medellín 050001, Colombia

**Keywords:** placenta, human placental explants, explant culture, histological analysis, full-term placenta (source: MeSH NLM)

## Abstract

Human placental explants (HPEs) culture has generated significant interest as a valuable in vitro model for studying tissue functions in response to adverse conditions, such as fluctuations in oxygen levels, nutrient availability, exposure to pathogenic microorganisms, and toxic compounds. HPEs offers the advantage of replicating the intricate microenvironment and cell-to-cell communication involved in this critical and transient organ. Although HPEs culture conditions have been extensively discussed, a protocol for assessing the viability and function of HPEs during short-term culture has not been previously outlined. In this study, we have developed a short-term HPEs culture protocol, specifically up to 72 h, and have employed quantitative, semi-quantitative, and qualitative analyses to evaluate tissue viability and function over time. Under our standardized conditions, placental villi explants began to regain their structural properties (the integrity of the trophoblast and villous stroma) and the functionality of the HPEs (production of angiogenic, endocrine, and immunological factors) starting from 48 h of culture. This restoration ensures a suitable environment for several applications. The data presented here can be highly valuable for laboratories aiming to implement an HPEs model, whether in the process of standardization or seeking to enhance and optimize working conditions and timing with placental tissue.

## 1. Introduction

The placenta is a transient organ that plays a crucial role in fetal development and growth. It consists of both fetal and maternal tissues and serves multiple functions, including the exchange of respiratory gases, nutrients, and waste products between the mother and the fetus, the synthesis of hormones and growth factors vital for a successful pregnancy, and the immune protection of the fetus. Disruptions in placental development and function have been associated with pregnancy complications and adverse outcomes, such as premature birth and low birth weight [1].

**Figure 1:**
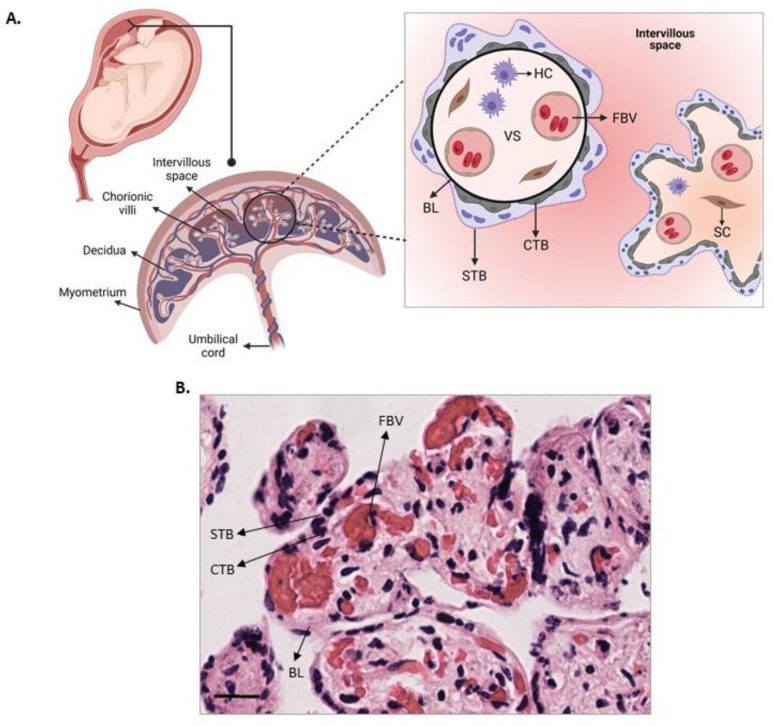
**Maternal–fetal interface and placental barrier.** (**A**) To the left is the placental tissue and fetus; to the right is the placental villus with a multinucleated layer of STB and an underlying mononuclear layer of CTB. STB cells cluster together, and they can form nodes as part of the final stage of the cell cycle of epithelial differentiation. The FBV are positioned close to the trophoblast. Compartments of placental tissue: decidua, villus, and intervillous space; CTB: cytotrophoblast; STB: syncytiotrophoblast; BL: basal lamina; FBV: fetal blood vessels; VS: villous stroma; HC: Hofbauer cells; SC: stromal cells. Illustration created with a BioRender license. (**B**) Cross-section of human placental explants stained with H&E, identifying the different regions and cells in the scheme in (**A**). Scale bar: 20 µm. Magnification 400×.

The fetal portion of the human placenta is organized into a branching network of villi. Floating villi are directly in contact with the maternal circulation and serve as the functional units of this organ [2]. Each villus is surrounded by a bi-layered epithelial barrier consisting of a multinucleated syncytiotrophoblast (STB) and the underlying mononucleated cytotrophoblast (CTB) (Figure 1). The STB layer undergoes continuous remodeling through the proliferation and differentiation of CTB cells into STB cells. The villi stroma is formed by connective tissue containing stromal cells, such as fibroblasts, fetal macrophages (Hoffbauer cells), and fetal vessels [3]. The cell layers that separate maternal blood from the fetal vessels are referred to as the placental barrier. In humans, the placental barriers include the STB, CTB, trophoblast basal lamina, cellular and extracellular components of connective tissue, and the endothelium of the fetal capillaries [3].

Considering the lack of an animal model that accurately replicates the anatomy and function of the human placenta, several in vitro systems have been developed to investigate the functionality of this organ. These systems include explant culture, primary cultures of placental cells (both CTB and STB), transformed trophoblast cell lines, and organoids [4]. In vitro models represent an invaluable tool for studying the pathogenesis of various diseases, as well as therapeutic options, complementing in vivo studies. Isolated CTB cells in primary culture could provide a useful system for studying individual trophoblast dynamics over the course of pregnancy; however, it has been argued that these approaches face disadvantages, including their isolation from significant interactions with other components of the villous structure [5]. 

On the other hand, human placental explants (HPEs) obtained from early and term placentas preserve various cell types in addition to the villous trophoblast and maintain the cellular architecture found in vivo. Since trophoblast function is influenced by interactions with other cells within the tissue, such as the physiological system, the culture of HPEs has proven to be a valuable model for studying nutrient transport, hormone production and release, secretion of other components, cell interactions, proliferation, and differentiation. This model is useful not only for investigating normal conditions but also for studying pathological processes and responses to infectious agents and xenobiotic insults [6]. Therefore, the culture of human tissues ex vivo allows the study of normal and pathogenic processes in the context of tissue cytoarchitecture under controlled laboratory conditions [7].

It is widely recognized that HPEs can be cultured for periods ranging from 7 to 11 days while maintaining stable tissue viability and endocrine function [5,6,8,9,10,11,12]. Consequently, although several published studies have described maintaining HPEs ex vivo, important technical details remain scattered, such as the proper time in which the HPEs are functionally and structurally optimal for conducting bioassays aimed at exploring their response to specific external stimuli, whether they be physical, chemical, or biological. 

This report offers a detailed protocol to culture human placental explants over a short period of time (up to 72 h), providing a viable third-trimester villous explant model suitable for investigating responses to external stimuli across a broad spectrum of biological research purposes. The standardization of short-term HPEs culture is important due to methodological and economic considerations. Minimizing the culture period duration, regardless of tissue origin, reduces handling and contamination risks, ultimately reducing the associated costs of the process.

Tissue dissection and manipulation should be performed gently and quickly to avoid tissue degeneration and detachment of the syncytiotrophoblast in the placental explant culture. The entire procedure must be performed under strictly sterile conditions to keep the chances of contamination at a minimum and should be complemented with the evaluation of the integrity and functionality of the villous trophoblast [6]. For the assessment of integrity and function, histological reading parameters, evaluation, and analysis of tissue damage and viability are presented, and tissue functionality is assessed through the production of angiogenic factors and cytokines.

## 2. Materials and Methods

The methodology is described in two sections: the first section outlines the detailed protocol for obtaining and culturing the explant, while the second section describes the methods used to validate the integrity and function of the cultured explant.

### 2.1. Detailed Protocol of Placenta Villi Culture

- **Subjects and Samples:** The placentas were donated by pregnant women aged between 23 and 33 years old; the inclusion criteria were pregnant women without comorbidities like hypertension, preeclampsia, or diabetes, who did not take medication, and who underwent scheduled cesarean sections instead of delivery induction. These women delivered via cesarean section after reaching 37 weeks of gestation, and their babies were also in good health. Neither the pregnant women nor their children should have been actively experiencing infectious processes. Exclusion criteria included maternal, fetal, or placental pathologies. The research protocols adhered to the principles of the Declaration of Helsinki and received approval from the Bioethics Committee of the Institute of Medical Research, Faculty of Medicine, University of Antioquia (Acta No. 015, dated 24 September 2020). All participants willingly participated in the study and provided informed consent by signing the documents approved by the Bioethics Committee. A total of twelve human term placentas (>37 weeks of gestation) were included in this study.
- **Collection of placentas from donors:** To collect the samples, the nursing staff from the Department of Gynecology and Obstetrics at Clinica El Rosario in Medellín provided the research team with a database containing candidates scheduled for cesarean surgical procedures who met all the inclusion criteria outlined in the Subjects and Samples section. Subsequently, these candidates were contacted by a member of the research team, who thoroughly explained the purpose of this study and requested their voluntary participation as placenta donors. Participation was confirmed through the signing of informed consent forms. Once the placenta, along with the umbilical cord, was delivered, it was immediately placed in a red bag and stored in an airtight plastic container. The placenta was then promptly transported to the laboratory (BSL II) to be processed within two hours following the cesarean delivery, as described below.

⚠**CAUTION:** Be sure to inform nurse personnel **DO NOT** put the placenta in formol.

⚠**CAUTION:** It is recommended that **NO MORE** than **2 h** pass between transport and processing to maintain trophoblast viability, and the tissue should be kept cold until processing.

### 2.2. Explants Culture

The culture of human placenta explants ex vivo to study the effects of external stimuli (i.e., infectious agents) included the following:

#### 2.2.1. Materials

- Hermetic box;
- Red biohazard disposal bags (Merck S.A., Darmstadt, Germany);
- Surgical tools: scalpel holder (Halomedicals Systems Limited, Port Harcourt, Nigeria); style scalpel blade (Fisherbrand™); high precision #10 dissection forceps (Thermo Fisher Scientific, Waltham, MA, USA); sharp-pointed dissecting scissors (Thermo Fisher Scientific, Waltham, MA, USA);
- Aluminum tray;
- Sterile glass Petri dishes VWR^®^;
- 6-well plates (Thermo Fisher Scientific^TM^);
- Blue cap bottle with blue lid, 500 mL (Sigma Aldrich, St. Louis, MI, USA);
- Pasteur pipettes 3 mL graduated (Thermo Fisher Scientific, Waltham, MA, USA);
- Serological pipette 10 mL graduated (Merck S.A., Darmstadt, Germany);
- Micropipette of 1000 mL and 200 mL graduated (Thermo Fisher Scientific, Waltham, MA, USA);
- Waste container;
- Pipette tips with multi guard barrier tips 100–1000 μL natural, sterile (Sorenson^TM^, Bioscience, Inc., Los Angeles, CA, USA) (Sigma Aldrich, St. Louis, MI, USA);
- Falcon™ 50 mL conical centrifuge tubes (Thermo Fisher Scientific, Waltham, MA, USA);
- Eppendorf microcentrifuge tubes 1,5 mL—clear color, safe lock caps, and DNA and RNA free (Bio-Seen) (Sigma Aldrich, St. Louis, MI, USA);
- Paper filters (Advantec A020F047A) (Sigma Aldrich, St. Louis, MI, USA).

#### 2.2.2. Reagents

- Antiseptic alcohol (ethanol 70%).
- Phosphate-buffered saline (PBS) 1× sterile 0780-50L (Sigma Aldrich, St. Louis, MI, USA).
- Antibiotic penicillin–streptomycin solution stabilized, with 10,000 units of penicillin and streptomycin/mL, 0.1 μm filtered, BioReagent, (Sigma Aldrich, St. Louis, MI, USA).
- Fetal bovine serum (FBS) F0926-500mL (Sigma Aldrich, St. Louis, MI, USA).
- Dulbecco’s modified Eagle’s medium/nutrient mixture F-12 Ham. Cell culture tested with L-glutamine and 15 mM of HEPES, without NaHCO_3_. Use at 15.6 g/L (DMEM/F-12) (Sigma Aldrich, St. Louis, MI, USA).
- Sodium bicarbonate-S6014 (Sigma Aldrich, St. Louis, MI, USA)
- 10% neutral formalin at room temperature 10%/ethanol 70% at 4 °C.
- Sodium hypochlorite solution (5000 ppm).

#### 2.2.3. Equipment

- Flow hood Nuaire Class II Type A2.
- CO_2_ Incubator Wiggens WCI-180.
- Inverted microscope Nikon Eclipse TS 100.
- Optical microscope Leica DM 500.
- Stereoscope Leica S6 with 1.25 × 20 zoom and a 60° viewing angle.
- Centrifuge Hermle Labnet Z 383 K.

#### 2.2.4. Protocol for Culture of Human Placental Explants

This section describes all the steps required to establish an optimal HPEs culture and to prepare placental villous in a short period of time for the study of external stimuli effects. This part includes the procedures conducted before, during, and after placental explant culture.

**Prior to placenta culture explants, the following steps were performed:**

- **Area disinfection:** ensure proper cleaning and disinfection of the safety cabinet using antiseptic alcohol.
- **Prepare a set of sterile instruments:** scissors, forceps, Pasteur pipettes, serological pipettes, and micropipettes.
- **Intra-cabin placenta container:** have an aluminum tray available that can hold the placenta when placed in the cabinet.
- **Culture dishes:** to culture the HPEs, new 6-well dishes are required; the number of dishes will depend on the researcher’s needs and objectives.

**Note:** leave the instruments numbered 1 to 4 under UV light inside the biosafety cabinet for at least 20 min.

- **Prepare culture dishes for seeding:** Before commencing the villus isolation process, add 1.5 mL of FBS to each well of the 6-well dishes and incubate at 37 °C for 2 h. After the incubation, remove 1.3 mL of FBS, leaving a thin layer where the HPEs will be seeded. (Note: be cautious not to wash too vigorously to prevent the removal matrix of nutrients from the bottom of the well).
- **Washing solution:** in a sterile glass bottle with a blue screw cap, combine 500 mL of filtered sterile 1× PBS with 5 mL of antibiotic.
- **Complete culture medium:** In a sterile 1000 mL bottle with a blue screw cap, reconstitute the F-12 Ham medium by adding 1.5 g of bicarbonate in distilled and sterile water until the total volume reaches 1 L. Mix for 30 min and then filter it using a vacuum system with paper filters. Next, transfer 45 mL of the prepared F-12 medium to a 50 mL Falcon tube. Add 5 mL of FBS (final concentration 10%) and 500 μL of antibiotics (final concentration 1×). Seal the Falcon tube with parafilm and store it at 4 °C. When using it, allow it to equilibrate to room temperature or warm at 37 °C for 10 min.

**Note:** the logistics for preparing the materials and workspace should be prearranged to prevent any delays during explants culture.

**Figure 2:**
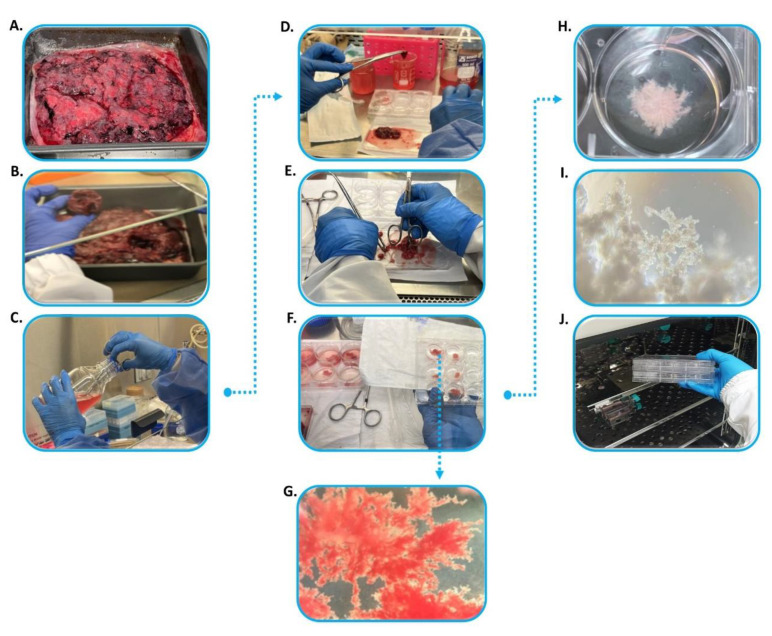
**Procedure for isolating human placenta villous.** (**A**) Placenta lying in an aluminum tray in a maternal portion of the surface facing upwards. At this point, the tissue should be weighed. (**B**) Small blocks of cotyledons located on the maternal side of the placenta are dissected, with dimensions of 3 × 3 cm and 2 cm in depth. Depending on the purpose of the study and tissue health, two or three cotyledons are required. (**C**) Each cotyledon is washed in a PBS 1X plus antibiotic (final concentration 1%) solution to remove blood clots. (**D**) Small fragments with dimensions around 4–6 mm are dissected from washed cotyledons. At this point, it is important to avoid capillaries during fragments dissection. (**E**) Villous from fragments are held using a blunt surface to press fragments until pieces with dimensions around 2–3 mm at most are obtained. (**F**) Additional washing of these small pieces is performed through immersion in well plates containing PBS plus antibiotic. Two or three pieces are washed together in a six-well plate, and then, the best tissues are selected for extra washing in a twelve-well plate. (**G**) Macroscopic appearance of the placental explant during washes under a stereoscope Leica S6 with 1.25 × 20 zoom and a 60° viewing angle. (**H**) After washing, villous are seeded in well containing a matrix of FBS for five to ten minutes. (**I**) Then, complete culture medium is added to the well plate to create a final volume of 3 mL; the placental villous are confirmed under optical microscopy. (**J**) The placental villous are cultured under standard conditions, with 5% CO_2_ at 37 °C for a period of 24 to 72 h, with daily medium changes.

**Culture of human placenta explants:** Figure 2 shows the principal steps for optimal HPEs culture in a short period of time for the study of external stimuli effects.

### 2.3. Procedure

- Inside a Biosafety Level II laboratory, remove the placental tissue from the red bag and place it on an aluminum tray for vigorous washing with tap water.
- Make sure that the fetal side along the umbilical cord is facing upwards; remove the amnion membrane by hands to obtain the cotyledons more easily and subsequently the chorionic villi.

**Note:** for the purposes of this protocol, the use of the umbilical cord is not required; so, a comprehensive processing and macroscopic analysis of it is not performed.

- Wash the maternal side of the surface containing cotyledons to remove large blood clots and necrosis areas using tap water. At this point, the delivered tissue should appear in good condition, having a ‘healthy’ pinkish color and lacking large blood clots and greenish necrotic centers (Figure 2A). The condition of the tissue is also dependent on how quickly it is delivered for processing [7].
- Introduce the washed tissue, placed on an aluminum tray, into a sterile laminar flow cabin for cotyledons dissection. The dissection can be performed manually, using a blade or scalpel, to obtain two or three cotyledons in different positions between the distal and central portions of the maternal surface. These cotyledons should have dimensions of around 3 × 3 cm and 2 cm in depth (Figure 2B). All cotyledons dissected must originate from healthy, pinkish region.

**Note:** the cotyledons should be obtained from a minimum of three different regions of the placenta, including sections located between the umbilical cord and the edge of the placenta, to ensure a representative sample of the tissue.

- Transfer the cotyledons to a glass jar containing a washing solution (sterile PBS 1× plus antibiotic 1%) (Figure 2C).
- Wash the cotyledons inside the Falcon tubes containing 50 mL of washing solution, performing approximately 10 inversions to thoroughly clean the tissue and remove any excess blood and clots that may have formed.
- Transfer the cotyledon into a 100 mm Petri dish containing 10–20 mL of washing solution to obtain the HPEs (Figure 2D). Dissect the cotyledon into small blocks fragments measuring around 4 mm. If capillaries are present, remove them using sterile forceps and scissors.
- Hold the villi using a blunt surface to prevent tissue breakage and obtain thin pieces with dimensions of approximately 2–3 mm at most (Figure 2E). Avoid dissecting larger pieces than ∼2 mm thickness; although they are easier to cut, their viability in culture can be compromised due to necrosis at their center, as a consequence of nutrient deficiency in the middle of the block [7].
- Remove erythrocyte excess by sequential washing the thin villous pieces in three wells of a six-well dish containing the washing solution (Figure 2F).
- During the washing process, perform a morphological inspection of the HPEs under a stereoscope to ensure tissue integrity (Figure 2G).
- A maximum of three placenta villi can be washed in the same well. At this point the villous should exhibit a light pink color (Figure 2H).
- To culture the HPEs, prepare the culture matrix in advance by adding 1.5 mL of FBS at the bottom of the culture wells and pre-incubate for 2 h at 37 °C.
- Following the matrix incubation, take out the six-well plate(s) from the incubator and remove 1 mL of the FBS added. Then, seed the HPEs (no more than three per well) and add 2 mL of complete F-12 (Figure 2H).
- Confirm the placental villi integrity under optical microscopy (Figure 2I) and culture under standard conditions, with 5% CO_2_ at 37 °C for a period of 72 h, with daily medium (F12 10% FBS) changes (Figure 2J). Make sure to remove the medium carefully by the walls of plates during changes to avoid releasing the adhesion layer formed by the trophoblast.
- Expose the placental villi to an external stimulus of interest. We recommend initiating treatments after 48–72 h of culture due to lower LDH activity (See Section 2.4.1.) and to ensure the prevention of bacterial or fungal contamination. Furthermore, this period is optimal for collecting supernatant samples to analyze metabolites of interest, such as LDH, hCG, other hormones, cytokines, and angiogenic factors.
- Dispose the remaining minced explants in a biohazard waste bag. All liquid waste should be collected in a beaker containing sodium hypochlorite solution and appropriately discarded.

⚠**CRITICAL:** due to biological variability among donors, the experimental design should involve donor-matched control and treatment groups, which means that each donor tissue should be divided into control and experimental groups.

For histochemical and immunohistochemical analyses, we recommend storing HPEs fragments in Eppendorf tubes with 10% neutral formalin at room temperature. We suggest that if immunohistochemical staining cannot be performed within the next 24 h after tissue storage in formalin, this tissue **SHOULD BE** transferred and stored in 1 mL of 70% ethanol at 4 °C to allow proper antigen retrieval for immunohistochemistry. 

If functional integrity assessment is necessary, it is recommended to preserve tissue fragments in Eppendorf tubes with 1 mL of Trizol for RNA extraction to evaluate the expression of inflammatory, angiogenic, and endocrine mediators, depending on the research objectives.

### 2.4. Methodological Approaches for Integrity and Function Validation

#### 2.4.1. Measurement of Viability through Lactate Dehydrogenase (LDH) Activity Detection

The activity of the LDH enzyme was measured as an indicator of cellular damage. The enzymatic activity of LDH was detected in culture supernatants using the “Cytotoxicity detection kit” (Roche Diagnostics GmbH, Mannheim, Germany). Briefly, after 24 h, 48 h, and 72 h of culture, 100 µL of the supernatant was transferred in triplicate to a 96-well plate. Subsequently, 100 µL of the kit’s reaction mixture (catalyst and dye solution) was added to each well. After 30 min of incubation in darkness at room temperature, the reaction was stopped by adding 50 µL of 2N sulfuric acid (R&D Systems, Minneapolis, MN, USA), and its optical density (OD) was measured at 450 nm using a microplate reader (Multiskan™ FC Microplate Photometer, Thermo Scientific™). Triton X-lysed tissue was used as a positive control for the technique, representing 100% of the LDH activity. 

#### 2.4.2. Detection of Endocrine Mediators and Angiogenic Factors to Assess HPEs Functionality

The functionality of HPEs was assessed through the detection of endocrine mediators and angiogenic factors, including the hCG hormone, Angiopoietin I (ANG-I), Vascular Endothelial Growth Factor (VEGF), Vascular Endothelial Growth Factor Receptor (VEGF R1/Flt-1), and Endoglin (END), by using the DuoSet ELISA enzymatic immunoassay kit (R&D Systems). These kits follow the sandwich ELISA principle and detect the free protein in culture supernatant. In brief, a 96-well microplate was coated with 50 μL of capture antibody overnight at room temperature. After three washes with buffer, the plate was dried with a clean towel. Then, the plate was blocked with 150 μL of blocking buffer and incubated at room temperature for one hour, followed by subsequent washes. Samples were added at a 1:1 (*v*/*v*) ratio with a final volume of 100 μL in each well and incubated for 2 h, followed by further washes. Subsequently, 50 μL of detection antibody was added to each well and incubated for 2 h with subsequent washes. Finally, 50 μL of Streptavidin-HRP solution was added per well for 20 min at room temperature, followed by the addition of 50 μL of substrate solution for 20 min at room temperature. The reaction was stopped with 50 μL of stop solution. The absorbances were read in a microplate photometer (Multiskan™ FC Thermo Scientific™) at 450 nm OD, and the concentration was determined in pg/mL by extrapolating the OD data into a standard curve.

#### 2.4.3. Histochemical Staining and Analysis 

HPEs samples were processed as follows: They were fixed in 10% formaldehyde in 0.1 M phosphate buffer (pH 7.3) for 24 h. Afterward, they were dehydrated in alcohol, clarified in xylene, embedded in paraffin, and sectioned into 3 μm slices. The paraffin-embedded tissue sections were subjected to different staining techniques for analysis: hematoxylin–eosin (H&E) staining for histological examination, which assessed the presence of syncytial knots, fibrin deposits, infarction, and total fetal blood vessels (as detailed in Table 1); Picro Sirius Red (PSR) staining; and Masson’s Trichrome (MT) staining for collagen histochemistry. For the analysis of the H&E-stained sections, the frequency of villi with the presence of different events was determined based on the total number of villi evaluated in 10 microscopic fields (Table 1). For the PSR analysis, the study followed established principles for valid histopathological scoring for research [13], as described in Table 2, adapted from [14]. Numerical values were assigned to the presence or absence of the event, (i.e., birefringence in PSR of the observed villi), and the average of these values was estimated across the observed villi in 10 microscopic fields. The slides were examined by light microscopy under 40× magnification (400-fold) and under 100× magnification (1000-fold, if the histological was needed to be confirmed). Note: as a control, the assessment of the integrity of fresh placental tissue through routine histological analysis using H&E staining could be included.

#### 2.4.4. Immunohistochemistry Staining and Analysis

The samples were processed using conventional methodology, which involved processes of deparaffinization in alcohol and xylene. Antigen retrieval was performed through incubation with sodium citrate buffer in a steamer for 30 min. The samples were incubated with the specific staining for trophoblast, using a polyclonal anti-CK-7 IgG antibody (GeneTex) 1:1000 diluted, and then, the immunocomplex were detected by a secondary antibody conjugated to peroxidase. The antigen–antibody complex was revealed using DAB chromogen, and nuclear contrast was achieved with Mayer’s hematoxylin. Negative control was established by replacing the primary antibody with phosphate buffer. Subsequently, 10 random fields were selected from each sample to be analyzed: detecting trophoblast detachment, denudation, and membrane rupture (Table 1). The frequency of different events was determined by the total number of villi examined across 10 microscopic fields. The slides were examined by light microscopy, under 40X magnification (400-fold).

**Note:** It is suggested to consider using additional markers alongside CK-7 to evaluate tissue integrity. These markers should be more specific to different types of cells present in the tissue, such as fibroblasts, vascular endothelium, and syncytiotrophoblast. By measuring these markers, it will provide a better understanding of the overall state of the tissue and its composition. This will offer more significant evidence of the model for evaluators who may use them.

#### 2.4.5. Evaluation of Apoptosis Using TUNEL Assay

To determine the frequency of apoptotic cells in HPEs, the DeadEnd™ Fluorometric TUNEL System was employed. This system is non-reactive and quantifies the fragmented DNA of apoptotic cells by catalytically incorporating fluorescein-12-dUTP(a) at 3′-OH DNA ends, utilizing the Terminal Deoxynucleotidyl Transferase, Recombinant (rTdT) enzyme [15]. In summary, 3-micron sections were cut from paraffin blocks using a microtome. These sections were subsequently mounted onto glass microscope slides. Once these mounts had dried, the slides were immersed in a 4% methanol-free formaldehyde solution in PBS (pH 7.4) for 25 min at 4 °C. Following this, the slides were washed by immersion in fresh PBS for 5 min at room temperature. The tissue sections were then permeabilized with 100 μL of Proteinase K for 5 min. After subsequent washing, 50 μL of equilibration buffer was added, and the slides were covered with plastic coverslips and incubated at room temperature for 5 min. Subsequently, the slides were incubated with 50 μL of the equilibration buffer containing the nucleotide mix and rTdT enzyme. The slides were covered with plastic coverslips and incubated at 37 °C for 1 h to allow the reaction to take place in a humidified chamber and protected from light. The plastic coverslip was removed, and the slides were dipped in 40 mL of 2× stop solution (NaCL + Sodium citrate) (1:10 in deionized water) and left for 15 min at room temperature. A final washing step was performed to remove any unincorporated fluorescein-12-dUTP. Finally, 50 μL of DAPI (Vector Lab Cat. # H-1200) nuclear stain in mounting medium was added and stored overnight at 4 °C in the dark. Afterward, the slides were washed, and the sample was analyzed to detect localized green fluorescence of apoptotic cells (fluorescein-12-dUTP) against a blue background (DAPI) using fluorescence microscopy. A standard fluorescein filter set was used to view the green fluorescence at 520 ± 20 nm. For data interpretation and comparison, a positive apoptosis control was used, consisting of HPEs treated with 20 ng/mL of TNF-α for 24 h.

#### 2.4.6. Measurement of Cytokines by Flow Cytometry

The levels of TH1/TH2/TH17 cytokine profiles were measured using the Cytometric Bead Array (CBA) Human TH1/TH2/TH17 Cytokine Kit (BD Bioscience). This assay provides a method to capture a set of analytes with known bead size and fluorescence, enabling the detection of analytes via flow cytometry. The cytokines measured were Interleukin-2 (IL-2), IL-4, IL-6, IL-10, Tumor Necrosis Factor (TNF), Interferon-γ (IFN-γ), and IL-17A. Briefly, 1:2 serial dilutions were prepared for eight points of the TH1/TH2/TH17 cytokine standard in a final volume of 300 μL. The assay diluent was used as a negative control. Next, capture beads for the seven cytokines were combined into a single vial along with the capture bead reagents (mixed capture beads). Vigorous mixing of the mixed capture beads was achieved using a vortex mixer. Then, 25 μL of mixed capture beads were added to each well of a 96-well plate. Subsequently, 25 μL of the standard, negative control and the samples were added to each well, followed by the addition of 25 μL of phycoerythrin (PE)-labeled TH1/TH2/TH17 detection reagent to each well. The samples were incubated at 4 °C overnight, protected from light. Next day, a washing step was performed using 500 μL of washing buffer per well, and the plate was centrifuged at 3290 rpm for 5 min. The supernatant was gently aspirated and discarded. Finally, 150 μL of washing buffer was added to each well, resuspended, and transferred to Falcon cytometry tubes for analysis. The cells were analyzed using a flow cytometry instrument (Cytoflex of Beckman coulter), and the data were analyzed using FlowJo v10.8.1 Software.

**Note:** The standard curve for each cytokine covers a defined range of concentrations. If you suspect that your samples have a high level of cytokines, it may be necessary to dilute the samples to ensure that their mean fluorescence values fall within the standard curve range.

### 2.5. Statistics

The experimental data were presented as the mean ± standard error of the mean (SEM). LDH, hCG, cytokines, and angiogenic factors released in the culture supernatants of the placental explants were normalized to tissue wet weight. A repeated measures test was employed to compare groups. A *p* value < 0.05 was considered statistically significant. The Tukey post hoc test was applied to compare different conditions. Graphs and statistical analyses were performed using GraphPad Prism version 10.

## 3. Results

### 3.1. Macroscopic Evaluation of Placental Tissue Integrity

**Figure 3:**
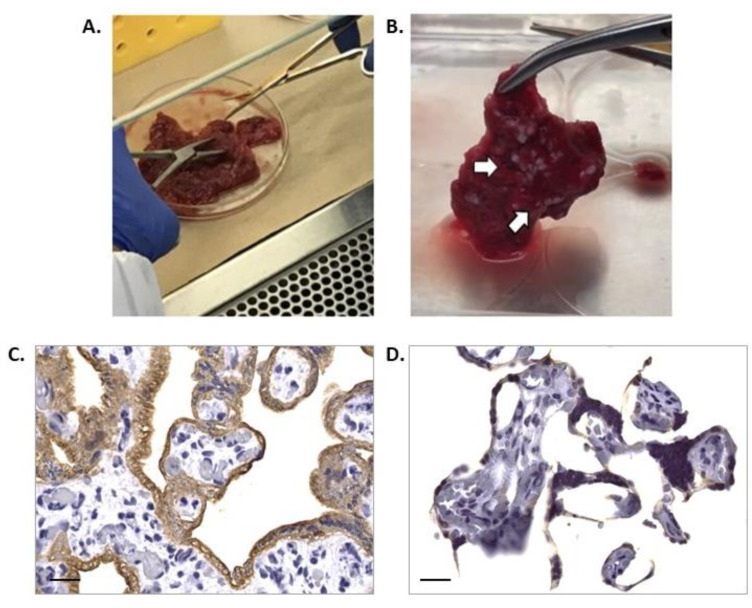
**Histological findings of HPEs that took more than 3 h to be processed.** (**A**) Macroscopic appearance of placental tissue in optimal conditions, ready to begin dissections and obtain the HPEs. (**B**) Tissue that took more than 3 h with poor macroscopic appearance that should not be used in culture (white arrows) and is not suitable for processing. (**C**) Cross-section of HPEs stained with CK-7, showing an intact trophoblast membrane with no evidence of detachment, rupture, or areas with denudation. (**D**) Cross-section of HPEs stained with CK-7, revealing a disruption of the trophoblast membrane, from tissue that took more than 3 h to process. Scale bar: 20 µm. Magnification 400×.

To illustrate the importance of processing time, there is evidence of macroscopic and microscopic histological events in HPEs that took more than 3 h to be processed. Tissue processed in a short period of time displayed healthy appearances without any signs of necrosis, infarction, or calcification (Figure 3A) and with normal histological conditions (Figure 3C). On the other hand, with a three-hour processing delay, macroscopic changes appeared, like the macroscopic appearance of the tissue, indicating an increase in coagulated, necrotic, and fibrotic areas (Figure 3B) Note: In addition to the processing time exceeding 3 h, suspicion of an unidentified or unrelated pathology from the clinical history should be considered, especially when the tissue’s appearance is like that shown in the photograph. Additionally, histological findings show alterations in the integrity of the trophoblast and villous stroma (Figure 3D). In some cases, the placenta may present macroscopic abnormalities such as calcifications, even if they originate from healthy pregnancies. In these cases, the placenta was discarded, and its processing was not continued. 

### 3.2. Microscopic Evaluation of HPEs Integrity

**Figure 4:**
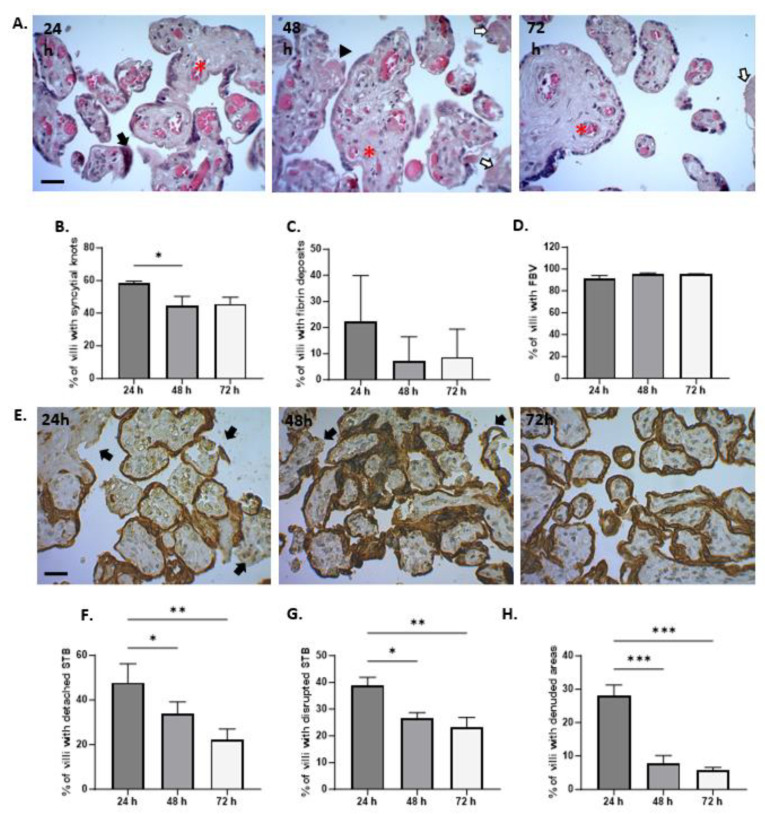
**Histological findings of HPEs in culture up to 72 h.** (**A**) Representative images of histological staining using H&E of tissue culture for 24, 48, and 72 h. Arrowhead: fibrin deposits, black arrow: syncytial knot, red asterisk: fetal blood vessels, white arrow: infarction. (**B**) Percentage of villi with syncytial knots, (**C**) percentage of villi with fibrin deposits, and (**D**) percentage of villi with fetal blood vessels. (**E**) Representative images of immunohistochemistry staining against cytokeratin 7 (CK-7) in tissue cultured for 24, 48, and 72 h. (**F**–**H**) The detachment, rupture, and denudation in HPEs cultured for 24, 48, and 72 h. One-way ANOVA of repeated measures with test of multiple comparisons (Tukey). (*) *p* value < 0.05, (**) *p* value < 0.01, (***) *p* value < 0.001. *n*: 6. Scale bar: 25 µm. Magnification 400×.

The morphological examination of the placental explants stained with H&E revealed that the tissue was mostly well preserved over the course of the culture period (Figure 4A). However, at 24 h of culture, syncytial nodules were significantly more frequent (58.33 ± 1.39), compared to 48 h (44.54 ± 5.81) and 72 h (45.71 ± 3.95) (*p* value 0.0316) (Figure 4B). Regarding fibrin deposits, no significant differences were found between the time points evaluated, but there was a positive trend observed at 24 h with a frequency of 22.27 ± 7.21, which decreased to 8.71 ± 4.35 at 72 h of culture (Figure 4C). The presence of fetal blood vessels within the villi stroma was detected at all time points, and the average frequency for this finding was similar at all time points, around 93% (Figure 4D).

Examination of the HPEs sections by immunostaining for CK-7 under culture conditions revealed degeneration of the STB layers within the first 24 h. During this time, detachment, rupture, and denudation of the trophoblast were significantly more frequent compared to 48 and 72 h after cultivation (Figure 4E). The frequency of detachment was 49.89 ± 8.29 at 24 h, which significantly decreased to 23.72 ± 3.32 at 72 h (*p* value =< 0.007) (Figure 4F). Similarly, the frequencies of rupture (Figure 4G) and denudation (Figure 4H) significantly decreased at 72 h of culture (*p* value = 0.0062 and *p* value =< 0.0001, respectively). This evidence suggests that, after 48 h, the tissue regenerates a new layer of trophoblast.

### 3.3. Organization of Collagen Fibers

**Figure 5:**
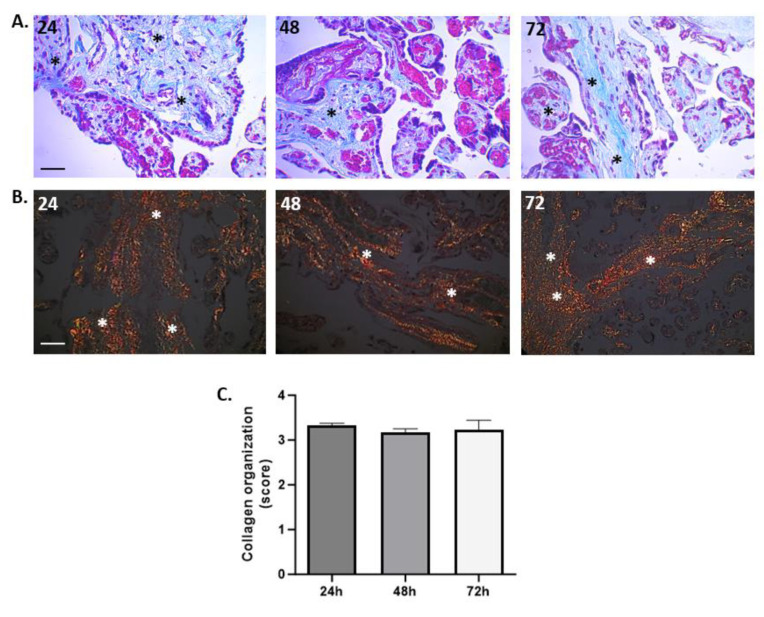
**The collagen organization fibers in villous stroma.** (**A**) Representative cross-sectional image of HPEs stained with Masson’s Trichrome (MT) stain. Qualitative analysis of the organization of collagen fibers type III in the villous stroma. Asterisks indicate collagen fibers (blue), red blood cells (red/magenta), and cell nuclei (purple/brown). (**B**) Photographic panel of cross-sectional HPEs stained with PSR; Asterisks indicate Col I in the villous matrix with PSR (in orange) is birefringent. (**C**) Statistical analysis of the data presented in B. There are no statistically significant differences in the collagen organization at 24, 48, and 72 h of culture. One-way ANOVA of repeated measures with test of multiple comparisons (Tukey). *p* value < 0.05, *n*: 3. Scale bar: 25 µm. Magnification 400×.

To assess not only the integrity of the trophoblastic barrier of the villi but also the structural integrity of the villous stroma, we employed Masson’s Trichrome (MT) staining (Figure 5A) and Picrosirius Red (PSR) staining (Figure 5B) to visualize the organization/distribution and arrangement of type IIII and I collagen fibers, respectively. Furthermore, the PSR staining was complemented by a semiquantitative analysis of birefringence in the stroma, as outlined in Table 2. Both the qualitative visualization of type III collagen fibers (Col III) and the analysis of type I collagen (Col I) allows us to conclude that the integrity of the villous stroma remained intact throughout the culture period. The average score frequency for assessing Col I birefringence with PSR staining was 3.12 ± 0.26 at 24 h and 3.28 ± 0.12 at 72 h. 

### 3.4. Tissue Viability and Endocrine Function in HPEs

**Figure 6:**
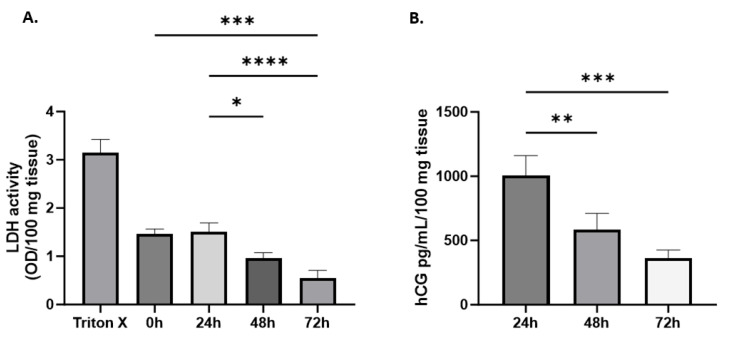
**The activity of lactate dehydrogenase significantly decreases throughout the culture, and the production of hCG decreases but remains steady up to 72 h.** (**A**) Lactate dehydrogenase (LDH) activity and (**B**) hCG production and release in the culture supernatant from placenta villous explants of term pregnancies, normalized by 100 mg of tissue. Data are means ± SEM from ten placentas (*n* = 10). One-way ANOVA of repeated measures with test of multiple comparisons (Tukey). (*) *p* value < 0.05, (**) *p* value < 0.01, and (***) *p* value < 0.001, (****) *p* value < 0.0001.

To investigate the viability of villous explants in culture, explants from 10 placentas were cultured for up to 72 h, and lactate dehydrogenase activity (LDH) and hCG production were measured in the culture supernatant every 24 h. The data suggest that placental explants can be cultured for up to 72 h with stable tissue viability, which becomes evident from 48 h onwards, along with evidence of placental hCG production. LDH measurement was also performed after 2 h of culture to establish a baseline representing the starting point of the culture and allowing for an understanding of the initial conditions. LDH values remained unchanged between 0 h and 24 h, but a significant decrease was observed at 48 h and 72 h (*p* value =< 0.0001) (Figure 6A).

The release of hCG into the culture supernatant was consistently observed, with a gradual increase at 24 h, resulting in an average hCG production of 1006 ± 155 pg/mL. This increase may be associated with the presence of maternal blood at the beginning of the culture, which decreases with successive washes in the subsequent cultures at 48 and 72 h, reaching a concentration of 364 ± 61 pg/mL at 72 h (Figure 6B).

### 3.5. DNA Fragmentation in HPEs Cells Using TUNEL Assay

**Figure 7:**
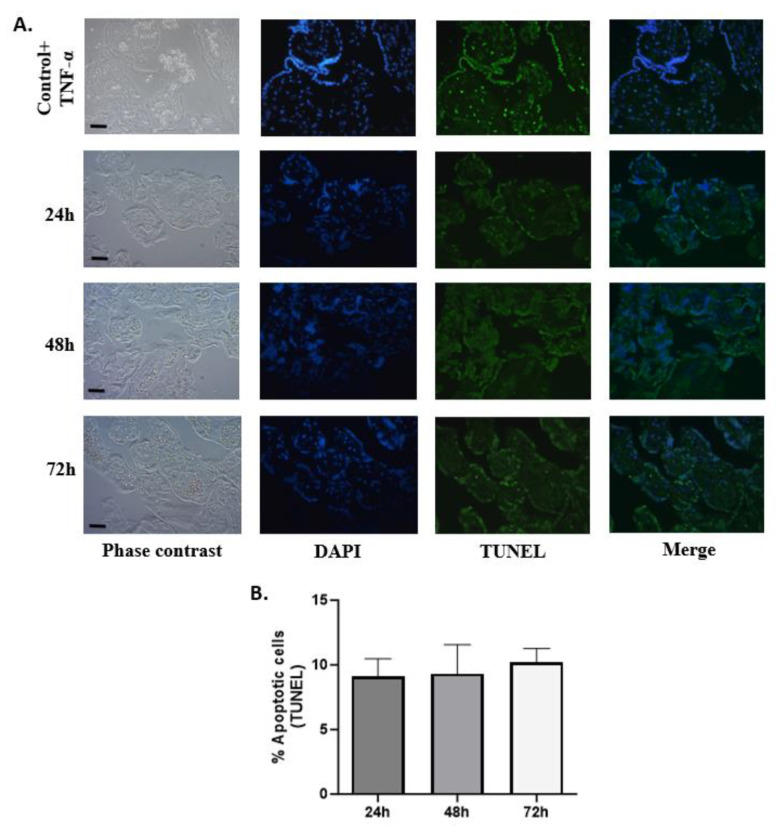
**Culturing time does not induce an increase in DNA fragmentation in HPEs.** (**A**) Representative photographic panel of HPEs in time kinetics labeled with TUNEL. The positive control corresponds to HPEs exposed to TNF-α (20 ng/mL) for 24 h. (**B**) Statistical analysis of the data presented in A. There are no statistically significant differences in DNA fragmentation at 24, 48, and 72 h of culture One-way ANOVA of repeated measures with test of multiple comparisons (Tukey). *n*: 3. Scale bar: 25 µm. Magnification 400×.

In situ labeling of fragmented DNA (TUNEL) assay was carried out to determine if the lower viability during the first 24 h of culture was due to apoptosis. The incidence of apoptotic trophoblast cells was low at all time points of culture analyzed, indicating that apoptosis was not the primary cause of cell death in cultured villous explants (Figure 7A). On average, the percentage of apoptosis remained around 10% and did not change at the three time points during the culture (Figure 7B).

### 3.6. Production and Release of Cytokines and Angiogenic Factors

The secretion of cytokines and angiogenic factors was monitored in the culture supernatant every 24 h because the dysregulation of these markers plays a role in the pathogenesis of certain diseases such as pre-eclampsia or in the response to infectious agents. The cytokine profile allowed us to determine whether this model is useful for studying the inflammatory environment following exposure to pathogens, drugs, and toxins, among other factors, under the culture conditions described in this protocol. IL-6 was produced in substantial amounts, with the highest levels observed at 48 h after culture, which remained stable until 72 h (Table 3). Secretion of IFN-γ was also detected, with no significant changes observed between the different culture time points, but with an upward trend by 48 h of culture. The production of IL-4, IL-17, IL-10, and IL-2 did not show any significant changes or defined trends between the different culture time points. However, it is worth noting that the cytokine that was produced the least in the HPEs was IL-2 (Table 3).

Regarding the evaluated angiogenic factors, the most produced factor was sFLT-1, followed by Endoglin, ANG-1, and VEGF. None of the studied angiogenic factors showed significant variations in their production relative to the culture time (Table 3).

The results of cytokine and angiogenic factor production in HPEs cultured for 24, 48, and 72 h are presented. Cytokines (*n* = 10); angiogenic factors (*n* = 7).

## 4. Discussion

This protocol provides an HPEs model derived from full-term placentas that have been cultivated for a shorter duration, thus reducing the typical cultivation times of 5 days [16] and 7 to 11 days [5] commonly observed in this type of culture. This allows for a quicker assessment, starting between 48 and 72 h. It is important to note that the time required for evaluating the effects of various bioassays on placental tissue can vary significantly and depends on the specific focus of each researcher, thus incubation periods may range from 2 h to several days. This protocol also considered including cotyledons from a minimum of three different regions of the placenta to ensure a representative tissue sample, a critical condition discussed and recommended by Roberts et al. [17], as the region from which the tissue sample is obtained can also have an impact on the results of functional tissue evaluation, such as cytokine production. 

Regarding the HPEs culture derived from full-term placentas, it should be noted that, unlike HPEs cultures derived from first and second-trimester placentas, they do not require a matrix for cultivation [6]. However, we obtained positive results using FBS as a matrix. This approach is both easy and economical, resulting in satisfactory outcomes. The integrity of the HPEs culture in FBS was confirmed at various time points throughout the protocol described in this study. A suggestion for researchers working with third trimester HPEs is to use this FBS matrix; it is relatively cost-effective, readily available in every cell culture laboratory, and user-friendly [6,18]. 

We evaluated the tissue integrity through macroscopic analysis before its inclusion and processing. During this evaluation, we ensured that tissue alterations such as coagulated, necrotic, and fibrotic appearances were not present. Macroscopic examination is crucial for distinguishing between what is considered a normal placenta and an abnormal one, which is why we recommend examining even normal placentas. Adapted guidelines from the College of American Pathologists suggest considering features such as masses, thrombi, and excessively long, short, or twisted umbilical cords in the macroscopic analysis of placental tissue [19].

The microscopic findings reported in this histological analysis study, such as syncytial knots and fibrin deposits, are typically found in HPEs cultures obtained from full-term placentas donated by healthy pregnant individuals. The increase in the frequency of syncytial knots at 24 h compared to 48 and 72 h may reflect the conditions of the tissue immediately after extraction from the in vivo environment, where the oxygen pressure at the time of delivery is low, around 40 mmHg [20,21], as opposed to the condition of the HPEs once it is subjected to stable oxygen conditions, around 95 mmHg, provided in the incubator at 48 and 72 h.

An increase in findings such as detachment, rupture, and denudation, evidenced by CK-7 labeling at 24 h, was observed, and this may be due to the tissue processing during the villi dissection step; so, experimental data intended for analysis during this time should be interpreted with caution. Other authors suggest that trophoblast detachment may be due to apoptotic processes that these cells typically undergo in epithelial turnover [22,23]; however, we did not observe changes in the TUNEL assay regarding cellular apoptosis at the different time points evaluated (24, 48, and 72 h). 

This increase in these findings may be attributed to the dissection process performed prior to the culture of HPEs, which inevitably may play a significant role in the integrity of the trophoblast membrane. Therefore, it is necessary to allow a minimum of 48 h of HPEs culture for the trophoblast membrane to regenerate, as evidenced by the decrease in the frequency of CK-7 findings over time. On the other hand, the constant replacement of a CTB membrane from STB allows dynamic changes in the trophoblast membrane to occur. This can manifest as detachment, ruptures, and denudations at different points of epithelial turnover [6]. However, in chronic processes and when there is no replacement of the trophoblast membrane, an increased frequency of these findings may be associated with pathological processes in placental tissue [24]. Previous studies have shown a progressive degeneration and viability loss of the original STB layers within the first 2 days of culture [5,25], but within 48–72 h, they could be regenerated by underlying viable cytotrophoblasts [5,26]. 

Monitoring the concentration of placental polypeptide hormones is an important part of the detailed characterization of explant cultures. The increase in hCG concentration for the same 24 h period can be explained by two reasons: contamination with maternal blood, even after thorough washing, but also tissue damage that may occur during the initial 24 h, resulting in greater intracytoplasmic release of hCG. Therefore, when LDH decreases, hCG also decreases, but there is always ongoing production.

It is essential to consider that when implementing a protocol using an HPEs model, significant inter-placental variations can occur [6,27]. An illustrative example of this phe-nomenon can be observed in the measurement of hCG production, where the maximum recorded value over 24 h reached 1720 pg/mL, while the minimum for the same duration was 361 pg/mL. However, the variability in hCG release data can be mitigated through the application of various normalization procedures. Therefore, it is advisable for each sample treatment to have its corresponding sample control. For instance, if a specific variable is to be applied to an HPEs derived from the placenta, identified by the code 'P01 treated,' an untreated counterpart should also be included to serve as its 'P01 control' This approach aims to minimize the influence of confounding factors resulting from data varia-bility that may arise during the experimental processes. 

We observed an expected immune response in explants derived from term-placentas from cesarean sections [28,29], characterized by elevated levels of IL-6 and reduced levels of TNF alpha, IFN-gamma, and IL-10 [30,31,32,33]. These results suggest the time frame proposed for HPEs culture in this study may be valuable for investigating cytokine expression in response to exogenous stimuli. Our results show an in vitro secretion of angiogenic pro-teins, mainly sFlt-1 and Eng. The higher production of sFLT-1 was expected, since previous reports suggest that at the time of full-term delivery, low oxygen pressure may induce an increase in sFLT-1 [34]. 

It is important to note that certain factors, such as the production of cytokines and steroid hormones, may undergo changes due to maternal medication use and labor induction [35]; one aspect to consider when assessing the immune response in the HPEs model is the potential triggering mechanism, especially in cases of natural childbirth compared to cesarean section. Factors such as the duration of labor and maternal treatment in the pre-ceding 48 hours may influence the inflammatory response and, consequently, the secre-tion of cytokines [36]. Therefore, recording maternal history is essential for the interpreta-tion of certain data. Another consideration to keep in mind for the interpretation of the functional assessment of placental tissue is the composition of the culture medium ac-cording to the parameters studied. If the laboratory where the cultivation of HPEs will be carried out uses different culture media than those used in the current protocol, it should be considered that the concentration of glucose can influence the production of placental growth hormone by explants [37]. 

It is crucial to highlight that, for researchers intending to implement this protocol, the minimal and most informative procedures for validating the viability, function, and in-tegrity of the culture are viability (LDH), function (hCG), and tissue integrity, specifically trophoblast integrity in this case (CK-7). Additionally, microscopic evaluation of the tissue could complement the analyses to validate the model. For example, factors such as LDH and hCG can be measured from the same 1 mL aliquot of tissue culture supernatant. On the other hand, certain steroid hormones such as estradiol and progesterone can be added to the measurements taken in this culture supernatant to complement the secretory func-tion of the tissue more comprehensively. This latter point represents a limitation in the current protocol, as these measurements were not included in this study. Nevertheless, researchers are encouraged to incorporate them into their procedures if feasible. Another important limitation to consider is that the integrity of tissue was not measured in freshly obtained placentas but rather starting from 24 hours of cultivation. Therefore, we encour-age researchers to guide their study by commencing with this control at time zero, in-cluding the placental tissue, to obtain more comprehensive information about the baseline conditions of their tissue.

In summary, we assessed the integrity and function of tissue samples dissected after 24, 48, and 72 h of culture. Histological evaluations of placental tissue indicated a restoration of integrity, as evidenced by a reduction in syncytial nodules and fibrin deposits, beginning at the 48-h mark after HPEs culture. The same trend was observed for trophoblast detachment, rupture, and denudation, which were more frequent during the initial 24 h but declined by the 48 h and 72 h time points evaluated. Furthermore, we observed a de-crease in LDH activity after 48 h of HPEs cultivation. In summary, all the variables studied in relation to crop viability and their function served in the present study to reach the conclusion that from 48 hours onward, the crop begins to be in optimal conditions for its use, and the choice of the time at which a specific condition is to be evaluated will depend on the researcher's objectives. 

## Figures and Tables

**Table 1:** Histological findings analyzed.

Histological Findings	Definition	Appearance (Scale Bar 25 μm)	Staining
Tissue infarction	The ischemic area of the villi due to the interruption of blood flow.	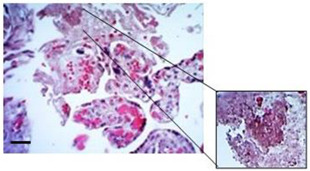	H&E
Syncytial node	Aggregates of syncytial nuclei on the surface of the villi.	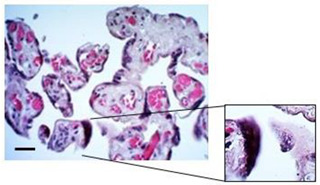	H&E
Fibrin deposits	Accumulation of fibrin in the stroma of the villus or around it.	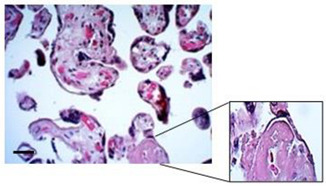	H&E
Capillaries	Number of capillaries per villus.	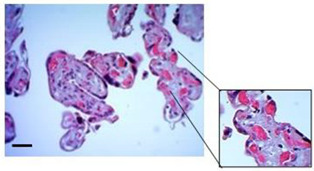	H&E
Membrane detachment	Represents a space observed between the trophoblast membrane and the villus; there is no complete union between them.	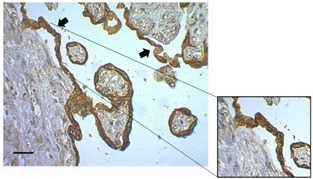	CK-7
Membrane disrupted	Represents a membrane break; loss of continuity of the trophoblast membrane.	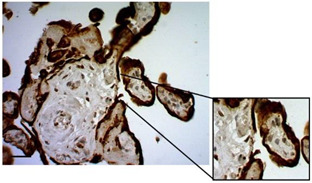	CK-7
Membrane denudation	Represents a villus partially or completely devoid of trophoblast membrane.	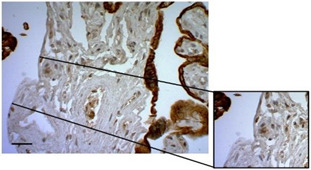	CK-7

Table summarizing the definitions of histological findings evaluated in cultured HPEs stained with H&E and CK-7. A zoom of each finding is shown for a better understanding of the description.

**Table 2:** Scores for the analysis of collagen organization in the villous stroma.

Score	Organization of Collagen I	Appearance
**1**	Absence of collagen birefringence	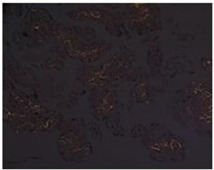
**2**	Low collagen birefringence	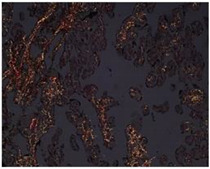
**3**	Moderate collagen birefringence	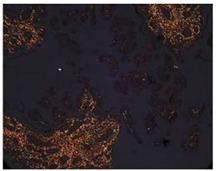
**4**	Strong collagen birefringence	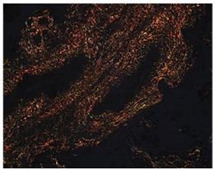

Score for the collagen organization analysis in the stroma of HPEs stained with PSR. Adapted from [14].

**Table 3:** Production and release of cytokines and angiogenic factors by human term placental explants in culture.

Cytokines [pg/mL]	24 h			48 h			72 h		
	Min.	Max.	Mean ± SEM	Min.	Max.	Mean ± SEM	Min.	Max.	Mean ± SEM
**IL-6**	2541	46,410	18,373 ± 4416	7504	4733	25,133 ± 4106	1974	47,314	25,067 ± 4244
**IFN-*γ***	47.2	41.5	44.6 ± 9.0	135.7	105.8	141.7 ± 6.9	79.25	70.29	72.19 ± 10.9
**IL-4**	40.1	115.3	69.2 ± 8.3	40.1	115.3	69.2 ± 8.3	40.2	115.4	69.2 ± 8.3
**IL-17**	26.0	68.9	43.6 ± 4.6	22.7	69.1	41.1 ± 5.0	23.3	70.8	42.7 ± 5.0
**IL-10**	4.5	18.9	8.5 ± 1.4	4.4	21.4	9.2 ± 1.7	3.4	16.5	8.7 ± 1.2
**IL-2**	4.2	12.2	7.0± 0.8	3.7	11.1	6.8 ± 0.8	4.0	11.8	6.9 ± 0.9
**Angiogenic factors** **[pg/mL]**									
**sFLT-1**	3574.0	11,275.0	7502 ± 998	3371.0	12,245.0	7490 ± 1147	2431.0	12,111.0	7013 ± 1143
**Endoglin**	32.01	98.7	62.9 ± 9.4	34.59	94.37	60.1 ± 8.7	30.57	105.2	58.9 ± 10.1
**Ang-1**	48.01	59.21	53.7 ± 4.2	48.12	57.61	51.5 ± 3.4	45.86	54.84	49.9 ± 3.5
**VEGF**	32.7	67.3	44.3 ± 7.8	35.3	88.5	59.8 ± 13.2	31.6	76.3	48.2 ± 9.8

## Data Availability

The authors confirm that the data supporting the findings of this study are available within the article.
